# Utilizing the Timed Up and Go Test to Predict Five-Year Mortalities Among Older Cardiovascular Inpatients: A Prospective Cohort Study

**DOI:** 10.31083/RCM37636

**Published:** 2025-08-21

**Authors:** Wenzheng Li, Min Zeng, Yuhao Wan, Jing Shi, Yaodan Liang, Ke Chai, Ning Sun, Wei He, Hua Wang, Jiefu Yang

**Affiliations:** ^1^Department of Cardiology, Beijing Hospital, National Center of Gerontology, Institute of Geriatric Medicine, Chinese Academy of Medical Sciences, 100730 Beijing, China; ^2^Beijing Hospital, National Center of Gerontology, Institute of Geriatric Medicine, Chinese Academy of Medical Sciences & Peking Union Medical College, 100730 Beijing, China; ^3^The Key Laboratory of Geriatrics, Beijing Institute of Geriatrics, Institute of Geriatric Medicine, Chinese Academy of Medical Sciences, Beijing Hospital/National Center of Gerontology of National Health Commission, 100730 Beijing, China

**Keywords:** Timed Up and Go Test, mobility function, older adults, cardiovascular disease, sarcopenia, prognosis, mortality

## Abstract

**Background:**

To examine the predictive value of the Timed Up and Go test (TUGT) for five-year mortality among older patients with cardiovascular disease (CVD).

**Methods:**

This prospective cohort study was conducted at the Beijing Hospital in China from September 2018 to April 2019, with a follow-up period of 5 years. Patients underwent the TUGT at baseline and were categorized into two groups based on the subsequent results: Group 1 (TUGT >15 s) and Group 2 (TUGT ≤15 s). The primary outcome of the study was all-cause mortality over five years.

**Results:**

The study included 491 older patients from the cardiology ward (average age 74.83 ± 6.38 years; 50.92% male). A total of 69 patients (14.05%) died over the five-year follow-up period. Patients in Group 1 were significantly older (78.36 ± 6.39 vs. 73.47 ± 5.83; *p* < 0.001) and exhibited higher prevalence rates of heart failure (HF) (21.17% vs. 11.86%; *p* = 0.009) and stroke or transient ischemic attack (TIA) (24.09% vs. 12.15%; *p* = 0.001) compared to those in Group 2. After adjusting for covariates, multivariate Cox regression analysis revealed that a TUGT >15 s in CVD patients was significantly associated with an elevated hazard ratio for five-year all-cause mortality (hazard ratio (HR): 2.029; 95% confidence interval (CI): 1.198–3.437; *p* = 0.004).

**Conclusions:**

The TUGT is independently associated with 5-year all-cause mortality among older patients with CVD, with a TUGT >15 s indicating a poorer prognosis.

**Clinical Trial Registration:**

ChiCTR1800017204; date of registration: 07/18/2018. URL: https://www.chictr.org.cn/showproj.html?proj=28931.

## 1. Introduction 

Global aging is accelerating, with an estimated 21% of the population projected to be over 65 by 2050 [1]. The prevalence of cardiovascular disease (CVD) increases with age, particularly for inpatients, resulting in high medical costs and significant healthcare burdens [2, 3]. Given the complexity and heterogeneity of health issues in older adults [4], it is essential to find a tool that can be widely applied to detect high-risk CVD patients. Mobility function is strongly linked to the overall health status of older adults [5, 6]. Furthermore, previous studies have shown that mobility impairment among CVD patients predicts an increased risk of mortality [7]. The Timed Up and Go Test (TUGT) is a simple, quick and cost-effective tool for quantitatively assessing mobility function. It is widely utilized for evaluating the mobility and balance of the older adults, patients, and individuals in rehabilitation [8]. Several studies have suggested that TUGT could be a useful tool to predict the risk for all-cause mortality in older adults [9, 10, 11]. However, there is still uncertainty about whether TUGT can predict outcomes specifically for older patients with CVD. This study aims to evaluate the performance of TUGT in predicting 5-year mortality among older inpatients with CVD in China.

## 2. Materials and Methods

### 2.1 Study Population 

The data for this study were derived from a prospective observational cohort study conducted in China (Trial registration: ChiCTR1800017204). Elderly patients aged 65 years and older, admitted to the Beijing Hospital between September 2018 and April 2019, were recruited. The criteria for inclusion were as follows: (1) Individuals aged 65 years or older, hospitalized due to cardiovascular illnesses; (2) voluntary participation in this study with signed informed consent. The exclusion criteria included: (1) patients unable to complete the TUGT and frailty assessment due to significant cognitive impairment, hearing loss, loss of mobility or other problems; (2) refusal to sign informed consent. The study received approval from the Ethics Committee of the Beijing Hospital (No. 2018BJYYEC-121-02).

### 2.2 Information Collection 

In this study, baseline data were obtained from the patients’ electronic health records. This data included information such as demographic information (e.g., age, sex), hospitalization details, medical conditions, physical examination results, laboratory test values, and other relevant health information. We utilized the Fried Frailty Phenotype (FFP) to assess baseline frailty in the patients. The FFP was widely used as a frailty assessment consisting of 5 criteria: unintentional weight loss, exhaustion, low grip strength, slow walking speed, and low activity. Patients with a score ≥3 were classified as frailty [12]. The FFP was assessed using standard protocols. Hand grip strength was measured using an electronic hand dynamometer (model EH101, CAMRY, Guangdong, China) (average of two dominant hand trials), with body mass index (BMI)-stratified cut-offs. Gait speed was determined by the faster of two 4-meter walks (with or without aids), with height-stratified cut-offs. Low physical activity was evaluated using the short Minnesota Leisure Time Activity Questionnaire, with thresholds of <383 kcal/week (males) and <270 kcal/week (females). All enrolled patients completed the full FFP assessment, with no exclusions based on feasibility, the specific diagnostic criteria are detailed in **Supplementary Table 1**. Based on the results of TUGT at baseline, participants were divided into two groups: Group 1 (TUGT >15 s), Group 2 (TUGT ≤15 s). A threshold of 15 seconds was used for grouping, as reported in previous studies [9]. Restricted cubic splines were employed to further validate the threshold associated with TUGT time and 5-year all-cause mortality.

### 2.3 Physical Function Test

TUGT was conducted at baseline by trained physicians or nurses. Meanwhile, it was generally conducted after the patient’s condition stabilizes (within 3–5 days of hospitalization) to ensure that the results reflect baseline functional mobility rather than transient influences such as acute heart failure. The standard procedure for the TUGT involves the patient performing a series of movements while being observed and timed. First, the patient starts by sitting in an armchair. The test begins when the patient rises from the chair, then walks a distance of 3 meters, turns around, walks back the same distance, and finally sits down again. The total time taken to complete this sequence is recorded [8].

### 2.4 Study End Point

The primary outcome of this study was the rate of all-cause mortality over a period of five years. Clinical follow-ups were routinely performed annually via phone. If the patient or their family was unable to be contacted, the patient’s medical records were used to determine their survival status. Because in China, nearly all individuals are covered by the national healthcare insurance system, we can verify and ensure the completeness of survival status by reviewing patients’ medical insurance records.

### 2.5 Statistical Analysis

Baseline continuous variables were summarized using either the mean and standard deviation (SD) or the median and interquartile range (IQR), depending on the distribution of the data. Categorical variables were presented as frequencies and proportions. For normally distributed continuous variables, Student’s *t*-test was used to compare data between groups. For non-normally distributed continuous variables, the Mann–Whitney U test was applied to compare data between groups. For categorical variables, the Chi-square test was utilized to compare data between groups. Restricted cubic splines were employed to investigate and visualize the relationship between TUGT time and 5-year all-cause mortality. Univariable and multivariable Cox proportional hazards analyses were conducted to identify factors associated with all-cause mortality over the 5-year period. Kaplan-Meier curves based on different TUGT groups were used to visualize the probability of survival over time. The log-rank test was applied to compare survival rates between these groups. In this study, variance inflation factor (VIF) values were computed for all predictors. A stepwise elimination procedure was applied, removing the variable with the highest VIF in each iteration until all remaining variables had VIF values below 5. To identify independent predictors of survival, Cox proportional hazards regression analysis was performed. A stepwise variable selection procedure based on Akaike’s Information Criterion (AIC) was applied using backward elimination. Starting from the full model, variables were sequentially removed to minimize the AIC value, aiming to achieve a parsimonious model. The final multivariable Cox model included covariates retained after stepwise selection. All statistical tests were two-tailed, and a *p*-value of <0.05 was considered statistically significant. All analyses were conducted using R software (version 4.2.2, R Foundation for Statistical Computing, Vienna, Austria).

## 3. Results

### 3.1 Baseline Characteristics

**Fig. 1.**
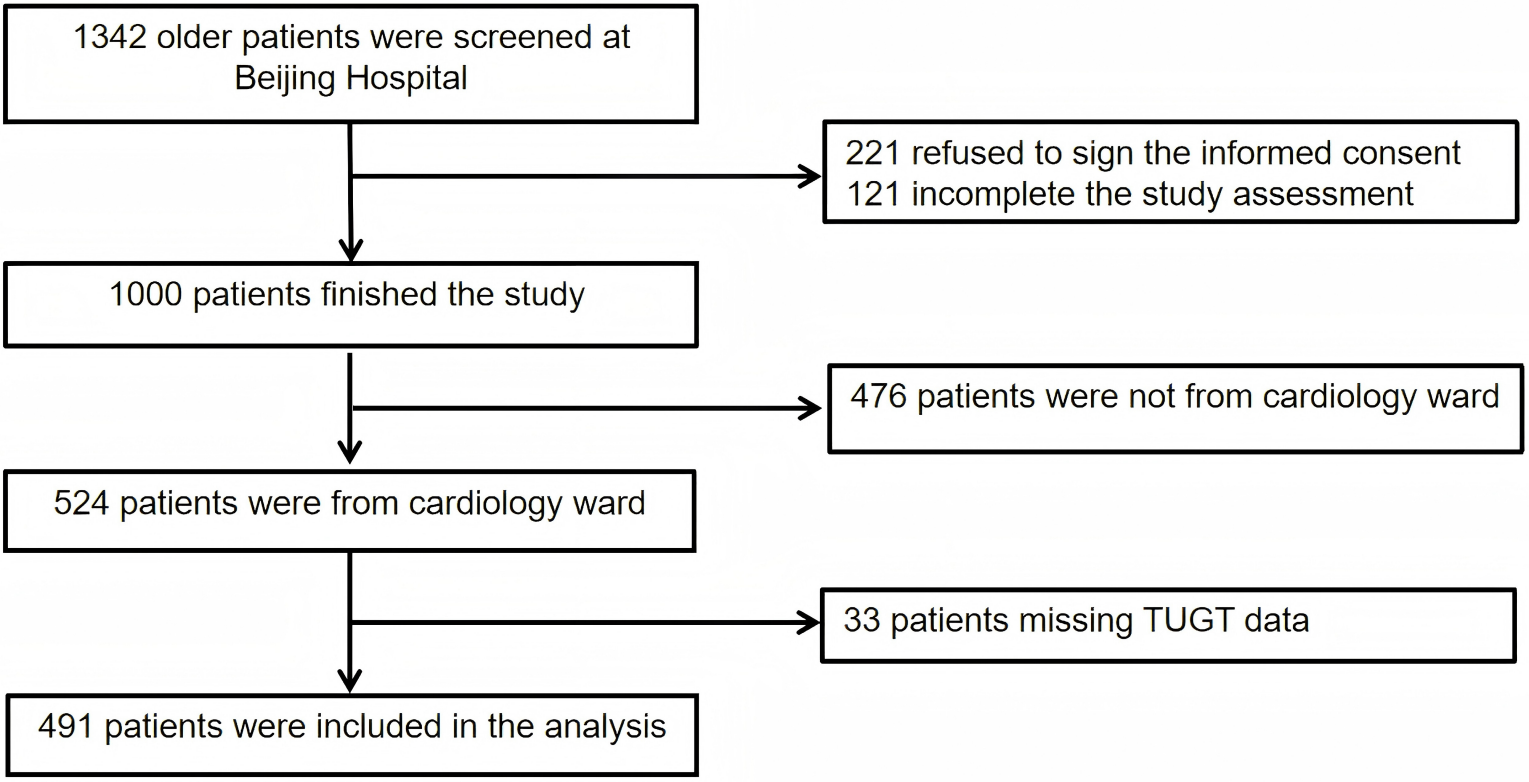
**Flow chart of patient selection**. Abbreviation: TUGT, Timed Up and Go Test.

A total of 491 older patients from the cardiology ward were included in the study. The participant inclusion process is illustrated in Fig. 1.

The baseline characteristics are summarized in Table 1. The mean age of participants was 74.83 ± 6.38 years, with 50.92% being males. Based on baseline TUGT results, participants were categorized into Group 1 (TUGT >15 s) and Group 2 (TUGT ≤15 s). Compared with the TUGT ≤15 s group at baseline, patients in the TUGT >15 s group were older (78.36 ± 6.39 vs. 73.47 ± 5.83, *p* < 0.001), had higher rates of heart failure (HF) (21.17% vs. 11.86%, *p* = 0.009) and stroke/transient ischemic attack (TIA) (24.09% vs. 12.15%, *p* = 0.001).

**Table 1.** **Baseline characteristics of patients by TUGT groups**.

	Overall	TUGT ≤15 s group	TUGT >15 s group	*p* value
	n = 491	n = 354 (83.5%)	n = 137 (16.5%)	
Age, y	74.83 ± 6.38	73.47 ± 5.83	78.36 ± 6.39	<0.001
Male, n (%)	250 (50.92)	188 (53.11)	62 (45.26)	0.119
BMI, kg/m^2^	25.32 ± 3.34	25.30 ± 3.21	25.36 ± 3.66	0.865
Hr, bpm	70.57 ± 13.37	69.43 ± 12.85	73.51 ± 14.25	0.003
SBP, mmHg	133.72 ± 16.84	133.22 ± 16.12	135.03 ± 18.58	0.286
DBP, mmHg	74.83 ± 9.51	75.15 ± 9.28	74.01 ± 10.06	0.236
AF/AFL, n (%)	109 (22.20)	72 (20.34)	37 (27.01)	0.111
CAD, n (%)	334 (68.02)	247 (69.77)	87 (63.50)	0.181
HTN, n (%)	355 (72.30)	253 (71.47)	102 (74.45)	0.508
CKD , n (%)	28 (5.70)	20 (5.65)	8 (5.84)	0.935
HF, n (%)	71 (14.46)	42 (11.86)	29 (21.17)	0.009
Stroke/TIA, n (%)	76 (15.48)	43 (12.15)	33 (24.09)	0.001
Diabetes, n (%)	169 (34.42)	127 (35.88)	42 (30.66)	0.275
Cancer history, n (%)	38 (7.74)	25 (7.06)	13 (9.49)	0.367
Frailty, n (%)	108 (22.00)	50 (14.12)	58 (42.34)	<0.001
HB, g/L	129.28 ± 15.43	130.34 ± 15.57	126.54 ± 14.78	0.015
LVEF, %	63.00 (60.00, 65.00)	65.00 (60.00, 65.00)	62.00 (60.00, 65.00)	0.143
NT-proBNP, pg/mL	167.70 (76.23, 415.55)	157.80 (69.09, 340.20)	280.20 (103.90, 970.70)	<0.001
5-year all-cause mortality	69 (14.05)	27 (7.63)	42 (30.66)	<0.001

Abbreviations: AF, atrial fibrillation; AFL, atrial flutter; BMI, body mass index; CAD, coronary artery disease; CKD, chronic kidney disease; DBP, diastolic blood pressure; HB, hemoglobin; HF, heart failure; Hr, heart rate; HTN, hypertension; LVEF, left ventricular ejection fraction; NT-proBNP, N-terminal pro-B-type natriuretic peptide; SBP, systolic blood pressure; TIA, transient ischemic attack. Continuous variables with a normal distribution are presented as mean ± standard deviation (SD), non-normally distributed continuous variables are expressed as median [25th–75th percentile], and categorical variables are shown as frequency (percentage).

### 3.2 Clinical Outcome

The 5-year survival status was available for all 491 participants, with all-cause mortality occurring in 69 patients (14.05%).

**Fig. 2.**
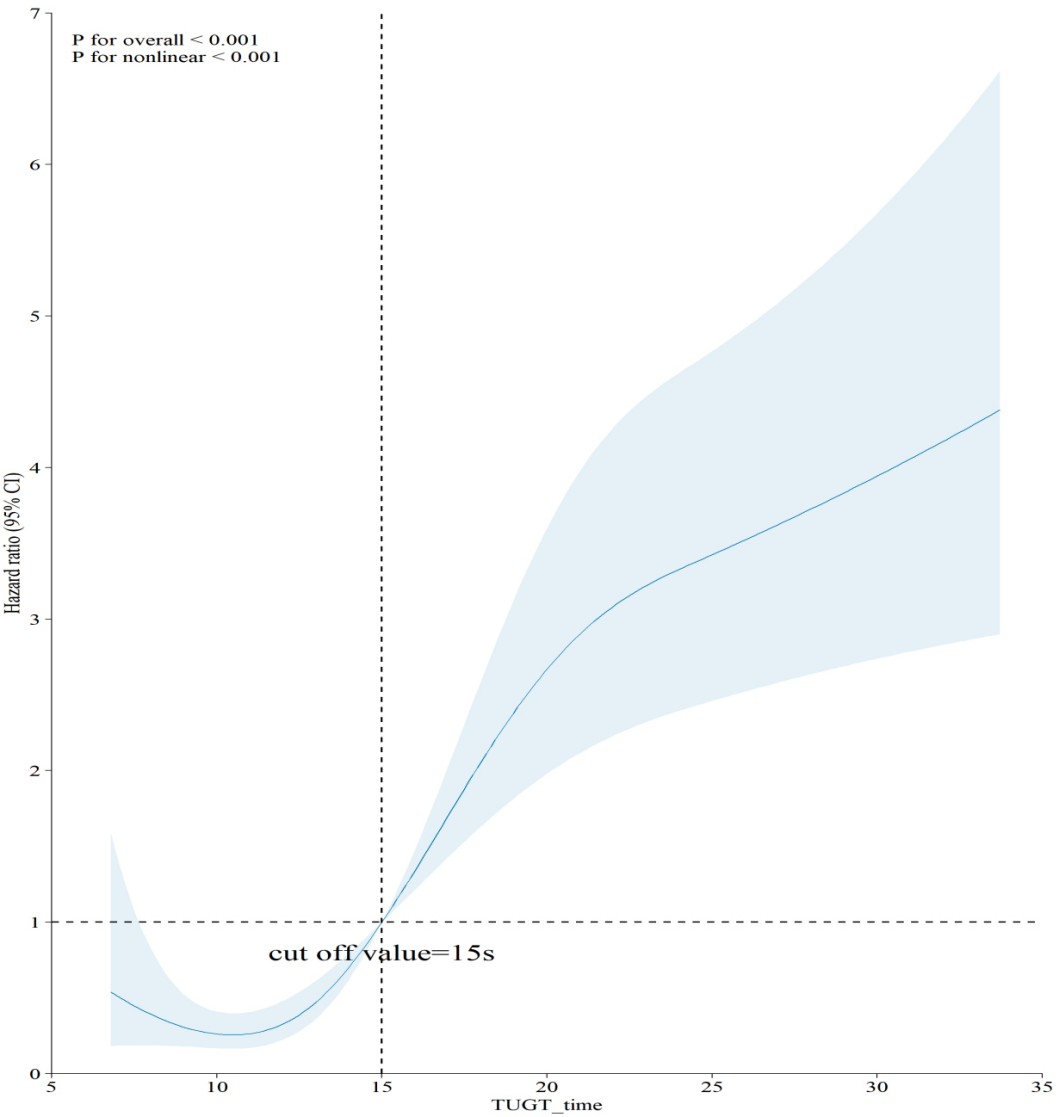
**Association of TUGT with 5-year all-cause mortality**. Abbreviation: TUGT, Timed Up and Go Test.

In Fig. 2, restricted cubic splines were utilized to explore and visualize the relationship between TUGT and five-year all-cause mortality. Prior research has consistently indicated that TUGT periods are primarily concentrated within the 10–20 second interval. In our analytical framework, the pivotal inflection point for mortality risk stratification was ascertained by pinpointing the time interval exhibiting the greatest slope gradient within this defined range, as calculated from the restricted cubic spline (RCS) curve. This method facilitated accurate measurement of the threshold at which incremental increases in TUGT length were most significantly correlated with heightened mortality risk. The analysis revealed a nonlinear relationship (*p* < 0.001), with the curve resembling an “L” shape. When TUGT was <15 s (on the left side), there was no significant increase in the hazard of all-cause mortality (hazard ratio (HR) <1). However, when TUGT was >15 s (on the right side), the hazard of all-cause mortality significantly increased (HR >1).

**Fig. 3.**
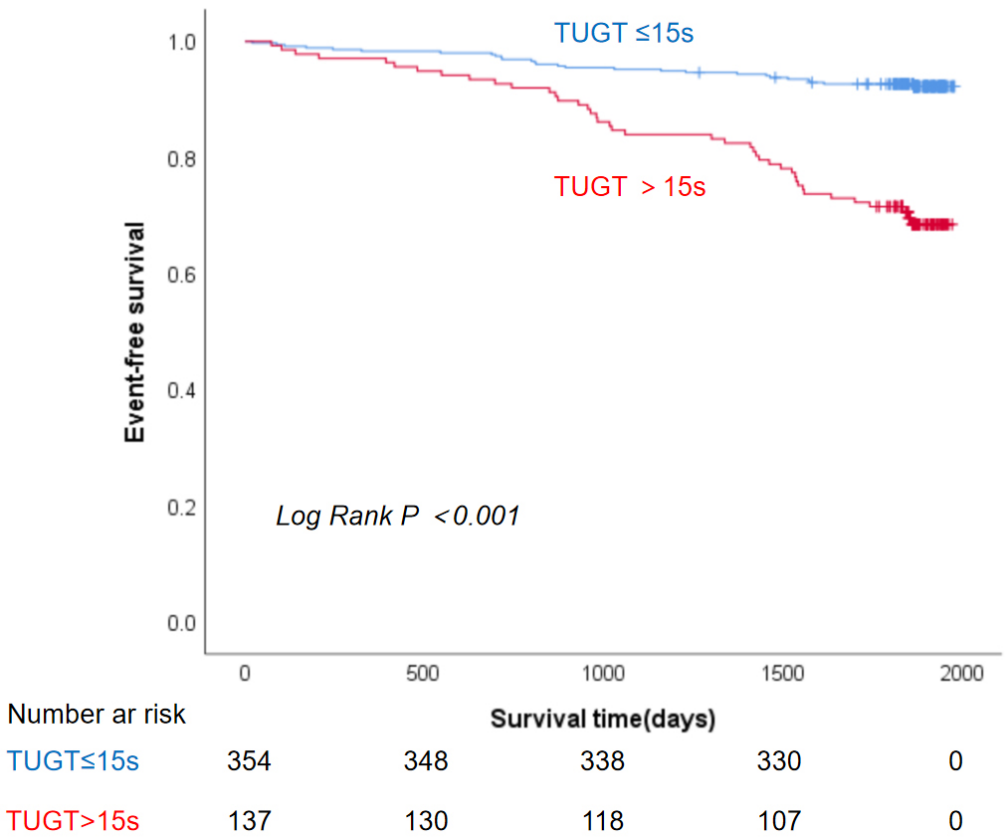
**Kaplan-Meier curve for the association between TUGT and 5-year all-cause mortality**. Abbreviation: TUGT, Timed Up and Go Test.

The Kaplan-Meier survival curve showed that patients with TUGT >15 s had a significantly higher all-cause mortality rate over five years (log-rank *p* < 0.001) (Fig. 3).

Univariable Cox regression analysis indicated that TUGT >15 s was a risk factor for all-cause mortality (HR: 4.510; 95% confidence interval (CI): 2.780–7.318, *p* < 0.001) (Table 2). In addition, aging, presence of frailty, chronic kidney disease (CKD), atrial fibrillation (AF)/atrial flutter (AFL) and elevated N-terminal pro-B-type natriuretic peptide (NT-proBNP) were identified as significant 5-year all-cause mortality risk factors in patients with CVD, whereas higher BMI, left ventricular ejection fraction (LVEF), and hemoglobin (HB) served as protective factors.

**Table 2.** **Univariable Cox regression analysis for 5-year all-cause mortality**.

Variables	Multivariable analysis		
	HR	95% CI	*p* value
TUGT >15 s	4.510	2.780–7.318	<0.001
Age	1.173	1.128–1.220	<0.001
Male	1.332	0.826–2.148	0.240
BMI	0.906	0.843–0.974	0.008
Hr	1.011	0.995–1.028	0.191
HF	4.430	2.731–7.188	<0.001
CAD	0.810	0.496–1.324	0.401
HTN	1.463	0.826–2.593	0.192
CKD	3.782	1.984–7.208	<0.001
AF/AFL	1.921	1.170–3.152	0.009
Diabetes	1.504	0.934–2.421	0.093
Stroke/TIA	1.274	0.697–2.330	0.431
Cancer history	1.967	0.976–3.963	0.059
LVEF	0.956	0.934–0.977	<0.001
HB	0.967	0.953–0.982	<0.001
LogNT-proBNP	3.987	2.587–6.144	<0.001
Frailty	4.580	2.854–7.349	<0.001

Abbreviations: AF, atrial fibrillation; AFL, atrial flutter; BMI, body mass index; CAD, coronary artery disease; CI, confidence interval; CKD, chronic kidney disease; HB, hemoglobin; HF, heart failure; Hr, heart rate; HR, hazard ratio; HTN, hypertension; Log NT-proBNP, Logarithm N-terminal pro-B-type natriuretic peptide; LVEF, left ventricular ejection fraction; TUGT, Timed Up and Go Test; TIA, transient ischemic attack.

Variables were ultimately selected for inclusion in the multivariable Cox regression model based on initial criteria, which included age, sex, and variables with a *p*-value < 0.10 in the univariate Cox regression analysis. Stepwise regression was then performed, and the AIC was calculated to select the optimal model. The initial variables included in the model were TUGT >15 s, age, male sex, frailty, BMI, AF/AFL, diabetes, cancer history, CKD, HF, HB, LVEF, and NT-proBNP. The initial AIC was 737.86. After stepwise regression, the final model included seven variables: TUGT >15 s, age, male, frailty, CKD, HB and HF, with an AIC of 729.43 and a concordance index (C-index) of 0.834. In the final model, TUGT >15 s was an independent risk factor for 5-year all-cause mortality (HR = 2.029, 95% CI = 1.198–3.437, *p* = 0.004). The final set of variables included in the model is shown in Table 3, along with the results of the multivariable Cox regression analysis.

**Table 3.** **Multivariate Cox regression analyses for 5-year all-cause mortality**.

Variables	Multivariable analysis		
	HR	95% CI	*p* value
TUGT >15 s	2.029	1.198–3.437	0.004
Age	1.129	1.081–1.178	<0.001
Male	1.435	0.871–2.362	0.031
HB	0.979	0.964–0.995	0.038
CKD	3.120	1.580–6.180	0.006
HF	2.565	1.556–4.227	<0.001
Frailty	2.350	1.436–3.846	0.001

Abbreviations: CI, confidence interval; CKD, chronic kidney disease; HB, hemoglobin; HR, hazard ratio; TUGT, Timed Up and Go Test; HF, heart failure.

## 4. Discussion

In this prospective cohort of older patients with CVD, a prolonged TUGT >15 s was found to be a significant independent predictor of increased all-cause mortality. After adjusting for age, sex, comorbidity and lab tests, the observed associations remained robust, suggesting that TUGT can be utilized as a predictive tool for 5-year clinical outcomes in hospitalized CVD patients.

Previous research (Table 4, Ref. [9, 13, 14]) has indicated that abnormal TUGT results are linked to an increased risk of all-cause mortality in community-dwelling older adults across diverse populations [9, 10, 11, 13]. A Peruvian study by Ascencio *et al*. [9] (2022) followed 501 adults aged 60 and older. The average follow-up period was 46.5 months. TUGT over 10 or 15 seconds was associated with lower survival rates. The strongest decline occurred when times exceeded 15 seconds. The study identified TUGT as an independent predictor of all-cause mortality, with each additional second increasing the risk of death by 5% (HR 1.05, 95% CI: 1.02–1.09).

**Table 4.** **Previous research about TUGT**.

First author	Year	Study objective	Study population	TUGT classification criteria	Study results
Chun [14]	2021	Evaluate the relationship between TUGT and the incidence of heart diseases and mortality	1,084,875 Korean 66-year-old adults (National Screening Program, 2009–2014)	<10 s, 10–19 s, ≥20 s	TUGT (≥20 s) is associated with increased MI, CHF, and all-cause mortality
Agnieszka Batko-Szwaczka [13]	2020	Evaluate the ability of the TUGT to predict adverse health outcomes in healthy aging community-dwelling early-old adults (aged 60–74), and to compare it with other functional measures like the frailty phenotype	160 community-dwelling adults from southern Poland (mean age 66.8 ± 4.2 years, 44.4% women), considered healthy aging early-old adults	≤9 s, >9 s	TUGT (≥9 s) demonstrated a significant association with elevated risks of adverse outcomes within one year
Edson J. Ascencio [9]	2022	Determine whether TUGT and Gait Speed can predict all-cause mortality	501 Peruvian older adults (mean age 70.6 years)	Two groups were compared using cutoff values of 15 s and 10 s, respectively	A prolonged TUGT correlates with reduced survival rates, particularly when TUGT exceeds 15 s

Abbreviations: CHF, congestive heart failure; MI, myocardial infarction; TUGT, Timed Up and Go Test.

A study was undertaken in Poland by Agnieszka Batko-Szwaczka and colleagues (2020) [13]. The study included 160 persons aged 60 to 74 residing in the community. TUGT ≥9 s was associated with increased chances of negative health outcomes within one year. The outcomes encompassed falls, hospitalizations, institutionalizations, or mortality. The TUGT outperformed the frailty phenotype in forecasting these outcomes. This benefit likely arises from its simplicity. The exam requires approximately 2.5 minutes for completion.

Meanwhile, Chun *et al*. [14] studied 1,084,875 community-dwelling 66-year-olds in Korea. They grouped TUGT as <10 s, 10–19 s, and ≥20 s. TUGT ≥20 s was linked to higher risks of myocardial infarction (MI), congestive heart failure (CHF), and all-cause mortality. Adjusted hazard ratios were 1.40 for MI, 1.59 for CHF, and 1.93 for mortality. The 10–19 seconds group had moderately increased risks. Our findings align with those of previous studies. Moreover, TUGT showed better performance in the Cox regression model 5 for predicting 5-year all-cause mortality, compared to single diseases or clinical parameters such as hypertension (HTN), AF, coronary artery disease (CAD), and LVEF, confirming that the predictive value of TUGT for all-cause mortality is applicable to patients with CVD.

CVD is one of the leading causes of death worldwide, contributing to a significant global health burden [15]. However, due to heterogeneity of CVD and the concurrent occurrence of multiple conditions in patients, a widely applicable and comprehensive tool or indicator for assessing the prognosis of CVD is lacking. The TUGT is a widely used assessment tool designed to evaluate functional mobility, balance, and risk of falls across various populations [8]. TUGT primarily reflects patients’overall physical performance and is not restricted to specific disease types, making it a promising tool for broad and comprehensive application in the prognostic assessment of CVD patients. Due to the simplicity, time efficiency, cost-effectiveness, and strong reproducibility of TUGT, our previous experience with this tool suggests that its application in CVD patients does not significantly increase the workload for healthcare providers. Patient safety is maintained during the testing process. Furthermore, TUGT can offer valuable insights for prioritizing targeted clinical follow-ups in high-risk CVD patients after discharge. In summary, our findings suggest that TUGT has significant potential risk stratification among older CVD patients.

We attempted to explain the mechanisms by which TUGT can predict clinical outcomes in older CVD patients. According to the European Working Group on Sarcopenia in Older People, 2nd Consensus (EWGSOP2), TUGT is utilized to categorize the severity of sarcopenia [16]. Sarcopenia, which is the progressive loss of muscle mass, strength and function, is a significant concern for older adults, particularly those with CVD [17, 18]. Sarcopenia is linked to an increased risk of several negative health outcomes, including death, falls, disability, hospitalization, and loss of independence [19]. There is a reciprocal relationship between CVD and sarcopenia [20]. Sarcopenia contributes to metabolic disturbances such as increased adiposity (fat), chronic inflammation and insulin resistance, which in turn increases the risk of CVD events [21]. Conversely, patients with CVD are often in a chronic inflammatory state, and conditions such as malnutrition and reduced physical activity can contribute to a catabolic state (where the body breaks down muscle and tissue). This accelerates muscle loss and worsens sarcopenia [22]. Aging itself brings about changes in body composition, including a decline in muscle and bone mass, an increase in body fat, and fat infiltration into muscles, bones, and organs like the liver. These changes lead to conditions such as myosteatosis (fat accumulation within muscle tissue) and sarcopenic obesity, where there is an increase in both fat and muscle loss [23]. Studies have shown that low lean muscle mass and sarcopenia are associated with higher arterial stiffness and arteriolosclerosis, which results in the thickening and hardening of small blood vessels [24, 25]. Inflammation, which is common in both sarcopenia and CVD, has also been linked to an increased risk of chronic diseases such as atherosclerotic CVD, HF, and adverse health outcomes [26]. Given the closely related mechanisms underlying sarcopenia and CVD, TUGT, as a measure of sarcopenia severity, has the potential to predict the outcomes of CVD disease in patients.

Our study indicates that TUGT can be an effective tool for early identification and screening of the mortality risk in older CVD patients. A systematic review of interventions targeting sarcopenia, such as exercise and nutritional strategies, found that most interventions improved sarcopenia-related measures (e.g., muscle mass and strength) [27]. Therefore, TUGT performance may also serve as a prognostic metric for patients undergoing nutritional and exercise interventions. Initiating TUGT assessments in middle-aged individuals may facilitate earlier detection of functional impairment and enable more timely intervention.

## 5. Strength & Limitations 

Our research especially focuses on high-risk older cardiovascular inpatients. Prognostic prediction in this group facilitates improved clinical decision-making, enhancing the applicability of the results in clinical practice. And based on our findings, we present a straightforward and user-friendly tool (TUGT) for forecasting long-term mortality risk in older cardiovascular patients, particularly those with heart failure and stroke/TIA. This offers crucial clinical utility for patient management. Moreover, our study utilized a prospective hospital-based design, ensuring detailed data and controlled conditions. The 5-year all-cause mortality endpoint was selected to capture long-term outcomes relevant to elderly CVD patients, where mortality rates are expected to be significant over this period.

Also, our study has some limitations. First, since this research was conducted at just one tertiary hospital with a relatively small sample size, the findings may not be generalizable to other populations or settings, which could limit their broader applicability. Second, the cohort was not specifically designed to investigate the relationship between TUGT performance and mortality risk. While a multivariate Cox regression model was used to account for potential confounding variables, there may still be residual or unknown confounders that could influence the results, potentially affecting the conclusions drawn from the data. Third, we utilized all-cause death instead of cardiovascular mortality as the outcome, we recognize the presence of competing risks in this population. This is an expansive term, and the etiology of certain fatalities may be unrelated to cardiovascular disorders or movement functions. Moreover, other confounding variables that could influence the correlation between physical performance and mortality were not considered in this study (e.g., sarcopenia, malnutrition, duration of hospital stay, drugs, etc.). Certain research indicate that polypharmacy may influence the mobility of older patients; nevertheless, our article did not address the patients’ medication usage [28].

## 6. Conclusions

TUGT demonstrated significant independent predictive value for 5-year all-cause mortality in older adults with CVD. A TUGT >15 seconds is associated with an elevated risk of mortality. TUGT offers advantages such as simplicity, non-invasiveness, and low cost, making it a useful tool for quick clinical assessment. These findings indicate that TUGT can be integrated into routine clinical practice to help healthcare providers identify patients who may benefit from more intensive monitoring and intervention.

## Availability of Data and Materials

Data are available upon reasonable request with the corresponding authors.

## Supplementary Material

Supplementary material associated with this article can be found, in the online version, at https://doi.org/10.31083/RCM37636.
