# Supplementary material for: Utilizing the Timed Up and Go Test to Predict Five-Year Mortalities Among Older Cardiovascular Inpatients: A Prospective Cohort Study
Source: Rev Cardiovasc Med. 2025 Aug 21;26(8):37636. doi: 10.31083/RCM37636 (PMC12415730; doi:10.31083/RCM37636)
Supplement: Supplementary file 1 [file 2153-8174-26-8-37636-s1.docx]

**Supplementary Table 1. Criteria of FFP**

|  | **Male** | **Female** |
| --- | --- | --- |
| Unintentional weight loss | >4.5kg over past year or >5% of previous year’s body weight | |
| Exhaustion | Any question ≥3 days / week | |
|  | 1. How often in the last week did you feel that you could not get going? | |
|  | 2. How often in the last week did you feel like everything you did was an effort? | |
| Decreased grip strength | Dominant hand grip strength measured by CAMRY electronic hand dynamometer for two times average | |
|  | BMI≤24kg/m^2^_，_≤29kg | BMI≤23kg/m^2^_，_≤17kg |
|  | 24<BMI≤28kg/m^2^_，_≤30kg | 23<BMI≤26kg/m^2^_，_≤17.3kg |
|  | BMI＞28kg/m^2^_，_≤32kg | 26＜BMI≤29kg/m^2^_，_≤18kg |
|  |  | BMI＞29kg/m^2^_，_≤21kg |
| Slow walking speed | The faster speed to walk 4 meters for two times, with or without a walking aid | |
|  | Height≤173cm_，_≥6.1s | Height≤159cm_，_≥6.1s |
|  | Height＞173cm_，_≥5.3s | Height＞159cm_，_≥5.3s |
| Reduced physical activity | Used the short version of the Minnesota Leisure Time Activity questionnaire | |
|  | <383kcal/week (walking 2.5h/week) | <270kcal/week (walking 2h/week) |
| 0 = Nonfrail ; 1-2 = Prefrail ; and ≥3 = Frailty | | |

Abbreviation: BMI, body mass index.
